# Self-assembly behaviors of thermal- and pH- sensitive magnetic nanocarriers for stimuli-triggered release

**DOI:** 10.1186/1556-276X-9-520

**Published:** 2014-09-22

**Authors:** Chih-Yu Kuo, Ting-Yu Liu, Andri Hardiansyah, Chia-Fen Lee, Man-Sheng Wang, Wen-Yen Chiu

**Affiliations:** 1 Institute of Polymer Science and Engineering, National Taiwan University, Taipei 10617, Taiwan; 2 Department of Materials Engineering, Ming Chi University of Technology, New Taipei City 24301, Taiwan; 3 Department of Materials Science and Engineering, National Taiwan University of Science and Technology, Taipei 10607, Taiwan; 4 Department of Cosmetic Science and Institute of Cosmetic Science, Chia Nan University of Pharmacy and Science, Tainan 71710, Taiwan; 5 Department of Chemical Engineering & Biotechnology, National Taipei University of Technology, Taipei 10608, Taiwan; 6 Department of Chemical Engineering, National Taiwan University, Taipei 10617, Taiwan; 7 Department of Materials Science and Engineering, National Taiwan University, Taipei 10617, Taiwan

**Keywords:** Tri-block copolymer, Poly(acrylic acid-b-[*N*-isopropylacrylamide]-b- acrylic acid), pH- and thermo- responsive polymer, Magnetic nanocarriers, Stimuli-triggered drug release

## Abstract

In the present work, we prepare thermo- and pH-sensitive polymer-based nanoparticles incorporating with magnetic iron oxide as the remote-controlled, stimuli-response nanocarriers. Well-defined, dual functional tri-block copolymer poly[(acrylic acid)-block-(N-isopropylacrylamide)-block-(acrylic acid)], was synthesized via reversible addition-fragmentation chain-transfer (RAFT) polymerization with S,S′-bis(α,α′-dimethyl-α″-acetic acid)trithiocarbonate (CMP) as a chain transfer agent (CTA). With the aid of using 3-aminopropyltriethoxysilane, the surface-modified iron oxides, Fe_3_O_4_-NH_2_, was then attached on the surface of self-assembled tri-block copolymer micelles via 1-[3-(dimethylamino)propyl]-3-ethylcarbodiimide hydrochloride/*N*-hydroxysuccinamide (EDC/NHS) crosslinking method in order to furnish not only the magnetic resources for remote control but also the structure maintenance for spherical morphology of our nanocarriers. The nanocarrier was characterized by transmission electron microscope (TEM), Fourier transform infrared spectroscopy (FT-IR), and ultraviolet–visible (UV/Vis) spectral analysis. Rhodamine 6G (R6G), as the modeling drugs, was encapsulated into the magnetic nanocarriers by a simple swelling method for fluorescence-labeling and controlled release monitoring. Biocompatibility of the nanocarriers was studied via 3-(4,5-dimethylthiazol-2-yl)-2,5-diphenyltetrazolium bromide (MTT) assay, which revealed that neither the pristine nanocarrier nor the R6G-loaded nanocarriers were cytotoxic to the normal fibroblast cells (L-929 cells). The *in vitro* stimuli-triggered release measurement showed that the intelligent nanocarriers were highly sensitive to the change of pH value and temperature rising by the high-frequency magnetic field (HFMF) treatment, which provided the significant potential to apply this technology to biomedical therapy by stimuli-responsive controlled release.

## Background

Over the past decades, polymeric nanoparticles have received great interests in drug delivery systems owing to their drug loading capacity and biocompatibility for targeting therapy [1]. As drug carriers, polymeric nanoparticles were able to load therapeutic agents, usually hydrophobic drugs, in the hydrophobic core region, while the hydrophilic shell region provided good dispersion in water and reduced the toxicity [2]. Polymeric nanoparticles also have shown prolonged-circulation characteristics and great-accumulation properties in tumor tissue because of the inefficient drainage by the lymph system, the so-called enhanced permeation and retention (EPR) effect [3]. Thus, efforts have been devoted to developing the stimuli-responsive polymers to be served as drug delivery vehicles, which could undergo a tremendous and reversible transition of morphology and dominate the precise controlled release, and work as a response to the various environmental stimuli, like temperature [4], pH [5], ionic strength [6], and magnetic field [7]. Thermo-sensitive polymers were widely investigated since the temperature was easy to control and could provide several advantages for *in vivo* and *in vitro* treatments. Among these thermal-sensitive polymers, poly(*N*-isopropylacrylamide) (PNIPAAm) was the best-known one; it preserved the considerable phase transition in aqueous system from hydrophilic to hydrophobic form around 32°C, which is the lower critical solution temperature (LCST) [8].

Since the LCST was close to the human body temperature, hydrophilic-segments, like acrylic acid (AA), were introduced into PNIPAAm for LCST tuning by co-polymerization [9], surface modification, or physical blending in order to prepare hollow structured nanoparticles via thermo-triggered self-assembling. Actually, the spherical sub-micron particle with empty core region possessed many advantages, for instance, higher drug loading content [10]. In spite of the benefits for drug delivery, the practical controlled release was still hindered with poor *in vivo* colloidal stability [11], which often caused low therapeutic efficiency and side effects.

On the other hand, magnetic materials were also widely used as nanocarriers' preparation because of the noncontact force which provided magnetically triggered release and remote treatment for drug delivery in therapy applications [12-17]. It was proved that the synergy-combined hyperthermia and chemotherapy was considerably effective for cancer therapy from the aid of tissue deoxygenating and cell-killing above 42°C [18]. Although magnetic sub-micron particles were the outstanding source as magnetic agent in the application of magnetic resonance imaging (MRI) [18], bioseparation, specific cell-detection, and so on, the applications of pure iron oxide (Fe_3_O_4_) were still limited due to its poor water dispersion. Thus, the incorporation or the surface modification of magnetic iron oxide with polymers has been employed to promote the biocompatibility and the drug-delivery capability.

In order to obtain the well-defined molecular-weight-distribution polymers, dual functional tri-block copolymer, P(AA-b-NIPAAm-b-AA), was synthesized by reversible addition- fragmentation chain-transfer (RAFT) polymerization in this research. The temperature-inducing self-assembling characteristic of the tri-block copolymers provide the excellence in drug encapsulation, owing to the interaction between hydrophobic PNIPAAm and hydrophilic poly(acrylic acid) (PAA) segments. Surface-modified iron oxide (Fe_3_O_4_-NH_2_) was introduced into the system via *N*-hydroxysuccinamide (NHS) and 1-[3-(dimethylamino)propyl]-3-ethylcarbodiimide hydrochloride (EDC) crosslinking method. Fe_3_O_4_-NH_2_ provided not only the stability of nanoparticles but also the ability of magnetic controlled release. After the encapsulated model drug, Rhodamine 6G (R6G), it gives the optical functionality via fluorescence labeling; the releasing behavior of R6G-loaded nanocarriers was studied under the condition of various temperatures and pH values. Furthermore, an external high-frequency magnetic field (HFMF) was applied for the remote control release of our intelligent magnetic nanocarriers.

## Methods

### Materials

*N*-Isopropylacrylamide (NIPAAm) was re-crystallized from n-hexane and acrylic acid (AA) was purified by vacuum distillation in order to remove the inhibitors before being used as the monomers. The RAFT chain-transfer agent, S,S′-bis(α,α′-dimethyl-α″-acetic acid)trithiocarbonate (CMP) was synthesized, following the protocol proposed by Lai and his coworkers [19]. Azobisisobutyronitrile (AIBN) was re-crystallized from methanol and then used as the thermal initiator. Rhodamine 6G (R6G), the model drug was purchased from Sigma-Aldrich. Iron(II) chloride tetrahydrate (FeCl_2_ · 4H_2_O) and iron(III) chloride (FeCl_3_) were used as the precursors of magnetic iron oxide (Fe_3_O_4_) nanoparticles. Ammonia solution (33%) was used as the reducing agent. 3-Aminopropyl triethoxysilane (APTES), NHS and EDC were introduced for surface modification. Over 99% of HPLC methanol was purchased from Sigma-Aldrich. Deionized water (18.2 MΩ) was used throughout the work. All chemicals and solvents were purchased from Sigma-Aldrich (Taipei, Taiwan), Acros (Taipei, Taiwan), or Fluka (Taipei, Taiwan) and used as received except otherwise noted.

### Synthesis of magnetic iron oxide nanoparticles (Fe_3_O_4_)

Magnetic nanoparticles, Fe_3_O_4_, were prepared by coprecipitation method as reported [20]. In brief, a mixture of FeCl_3_ and FeCl_2_ · 4H_2_O with 2:1 molar ratio was dissolved in deionized water. After completely dissolving, ammonium hydroxide was added dropwise under mechanical stirring at room temperature for 30 min. Then, the magnetic iron oxides were collected by magnet and washed with water three times.

### Surface-modification of magnetic iron oxide with amine groups (Fe_3_O_4_-NH_2_)

The sol–gel method was applied for the surface modification of Fe_3_O_4_ with amine groups. In short, Fe_3_O_4_ was dispersed with ammonia solution in an ethanol/water (9/1, *v*/*v*) solution at pH greater than 11 with sonication for 30 min. APTES was then added into the system and stirred for another 12 h at 25°C. Thereafter, the magnetic iron oxide nanoparticles were collected and re-dispersed in deionized water for further use.

### RAFT polymerization of tri-block copolymers P(AA-b-NIPAAm-b-AA)

AA monomer (3.062 g) was dissolved in 20 ml of methanol containing 0.12 g of chain-transfer agent, CMP, and 0.017 g of initiator, AIBN. After the chemicals were totally dissolved, the reaction was preceded for 3 h at 70°C and purged with nitrogen, so that the conversion of AA monomers could reach 99.29%. NIPAAm monomer (7.21 g) was dissolved in 20 ml methanol then introduced into the system for another 14 h and the tri-block copolymer P(AA-b-NIPAAm-b-AA) solution was obtained. The solution was first dialyzed with methanol so as to remove unreacted monomers, and then vacuum-dried at room temperature. Therefore, the P(AA-b-NIPAAm-b-AA) copolymer, A_100_N_150_, was collected. A_x_N_y_ represents the mole feeding ratio of *x* mole of AA and *y* mole of NIPAAm monomers to chain transfer agent (CTA) molecules.

### Preparation of self-assembled magnetic nanocarriers (A100N150/Fe_3_O_4_-NH_2_)

Fe_3_O_4_-NH_2_ was covalently bonded with the tri-block copolymer, P(AA-bNIPAAm-bAA) via the 1-[3-(dimethylamino)propyl]-3-ethylcarbodiimide hydrochloride/*N*-hydroxysuccinamide (EDC/NHS) method as described in the preparation of nanocarriers. A_100_N_150_ (0.25 g) was dissolved in 50 ml H_2_O, and the pH value was adjusted to 4.5 with the diluted NaOH solution. After the self-assembling process at 60°C for 2 h, 0.05 g of magnetic iron oxide, Fe_3_O_4_-NH_2_ was added into the system and then stirred for another 4 h at 60°C. EDC (0.2 g) and NHS (0.2 g) were then added into the solution for activation and chemical bonding at 60°C for another 24 h. The self-assembled magnetic nanocarriers, A_100_N_150_/Fe_3_O_4_-NH_2_, were centrifuged and washed with water three times and eventually collected as powder after lyophilization for further use.

### Preparation of drug-loaded nanocarriers with high-concentration R6G solution

A_100_N_150_/Fe_3_O_4_-NH_2_ (0.8 g) was dropped in a 30 ml aqueous solution which contained 300 mg of R6G, and the system was kept shaking for 24 h at room temperature for equilibrium. Swelling and hydrogen bonding led to the drug-loading process of magnetic nanocarriers. After centrifugation, the drug-loaded nanocarriers were collected and preserved at room temperature for further experiments. Meanwhile the encapsulation efficiency (EE) and drug loading content (DLC) were calculated per Equations 1 and 2

(1) $$\mathrm{EE}=\frac{\mathrm{weight}\;\mathrm{of}\;\mathrm{encapsulated}\;\mathrm{drug}}{\mathrm{total}\;\mathrm{weight}\;\mathrm{of}\;\mathrm{drug}\;\mathrm{in}\;\mathrm{feed}}$$

(2) $$\mathrm{DLC}=\frac{\mathrm{weight}\;\mathrm{of}\;\mathrm{encapsulated}\;\mathrm{drug}}{\mathrm{weight}\;\mathrm{of}\;\mathrm{nanocarriers}}$$

### Controlled release behavior

**Figure 1:**
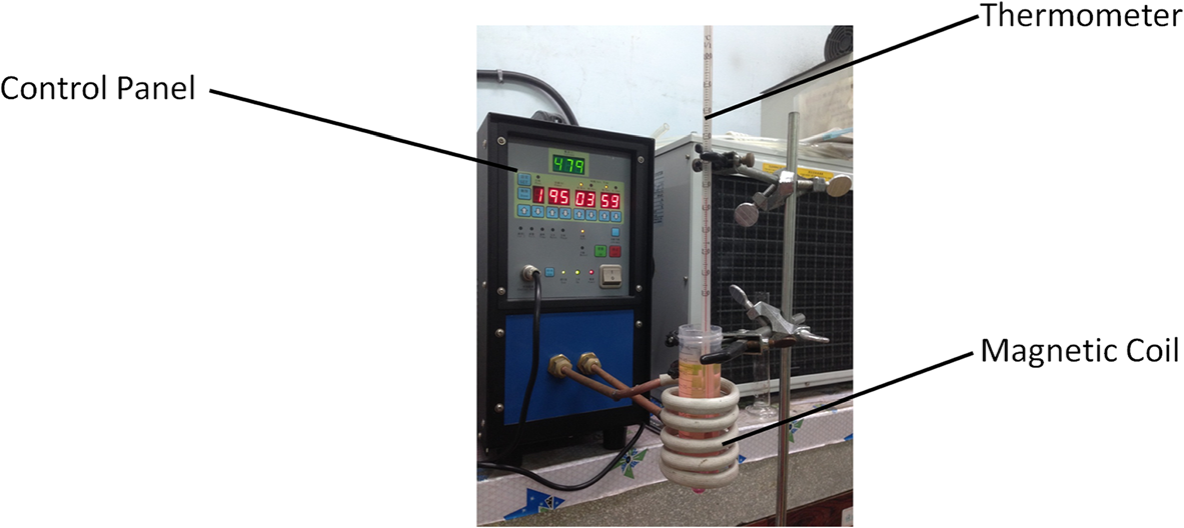
Experimental apparatus of magnetic-triggered release under high-frequency magnetic field (HFMF).

Various release environments were accomplished in the buffer solutions with different pH values and temperatures. R6G-molecule-encapsulated nanocarriers (20 mg), A_100_N_150_/Fe_3_O_4_-NH_2_/R6G, were placed in a dialysis bag (MWCO = 3,500). The dialysis bag was then immersed in 200 ml of buffer solution at specific pH values (3 and 7.4) and temperatures (25°C, 37°C, and 60°C). At the prescribed time interval, 1.5 ml of the buffer solution was withdrawn and replaced with the fresh medium. The concentration of the released R6G was determined by ultraviolet–visible (UV/Vis) analysis with the calibration curve of R6G for various concentrations in aqueous solution. Magnetic-triggered release was accomplished by radio heating generated by -HFMF- as shown in Figure 1, operating at 15 kHz. During the magnetic-field-triggered release, the temperature was recorded to measure the cell-killing effect due to the hyperthermia therapy. Meanwhile, some solution was withdrawn and its concentration of the released drugs was measured by UV/Vis at specific time intervals. The results of the drug-release experiment presented here indicate an average of at least triplicate measurements, and the release percentage was defined as the following equation.

(3) $$\mathrm{release}\;\mathrm{percentage}=\frac{\mathrm{weight}\;\mathrm{of}\;\mathrm{drug}\;\mathrm{released}\;\mathrm{in}\;\mathrm{the}\;\mathrm{buffer}\;\mathrm{solution}}{\mathrm{weight}\;\mathrm{of}\;\mathrm{drug}\;\mathrm{encapsulated}\;\mathrm{in}\;\mathrm{nanocarriers}}$$

### Molecular characterization

#### ***Gel permeation chromatography (GPC)***

The average molecular weight and molecular weight distribution of P(AA-b-NIPAAm-b-AA) via RAFT polymerization were determined by GPC, which was equipped with a solvent delivery system, two columns (Malvern Viscotek T-columns LT5000, Malvern Instruments, Ltd., Taipei, Taiwan) maintained at 40°C, and a differential refractometer (RI). Sodium sulfate aqueous solution (0.025 M) was applied as the eluent at a flow rate of 0.8 mL/min. Poly(methylacrylic acid) standards were used for calibration standard and the molecular weight was reported. A diluted sodium hydroxide solution (0.02 g) was also used to improve the solubility of tri-block copolymer in 4 ml of sodium sulfate aqueous solution.

#### ***Nuclear magnetic resonance spectroscopy (NMR)***

NMR was applied to determine the ratio of two monomer units (AA and NIPAAm) in P(AA-b-NIPAAm-b-AA). Therefore, ^1^H NMR spectra were recorded on a Bruker AV-400 NMR spectrometer (Bruker, Taipei, Taiwan), using D-methanol (D4) or D-chloroform (D1) as solvent.

#### ***Fourier transform infrared spectroscopy (FT-IR)***

FT-IR absorption spectra were recorded by PerkinElmer Spectrum 100 spectrometer (PerkinElmer, Taipei, Taiwan) at frequencies ranging from 400 to 4,000 cm^-1^ with 4 cm^-1^ resolution. The samples were mixed with KBr and pressed into pellets.

#### ***LCST of tri-block copolymer, P(AA-b-NIPAAm-b-AA)***

The cloud point or LCST of P(AA-b-NIPAAm-b-AA) was measured by using a UV/Vis spectrophotometer (Thermo Spectronic Gamma Series, Thermo Scientific, Taipei, Taiwan). Latex solutions were prepared by dissolving 120 mg of samples in 4 ml of different pH value solutions. Then, the transmittance of the solution at 480 nm was detected with the heating rate of 5°C/min at different temperatures.

### Zeta potential measurement

The surface potential of self-assembled sub-micron particles was measured via zeta potential analyzer (Zetasizer Nano, Malvern Instruments Ltd., Taipei, Taiwan). The zeta potential data reported were the average values of triplicate samples with standard deviation.

### Morphology observation

The latex solutions were diluted with deionized water and dripped on a copper grid coated with a collodion. It was dried in air or in an oven with specific temperatures and observed by using JEOL JSM-1230 transmission electron microscope (TEM) (Jeol, Taipei, Taiwan).

### Cell lines and cytotoxicity assay

L-929 fibroblast cells were purchased from the Food Industry Research and Development Institute (FIRDI, Hsinchu, Taiwan) and was cultured using Dulbecco’s modified Eagle's medium (DMEM) composed of 10% (*v*/*v*) fetal bovine serum (FBS) and 1% (*v*/*v*) antibiotic antimycotic solution. The cell lines were incubated under saturated humid conditions at 37°C and 5% CO_2_, and the medium was changed every 1 to 2 days until confluence was reached at approximately 70% to 80% confluency.

Cytotoxicity analysis of the magnetic nanocarriers was determined via the standard 3-(4,5-dimethylthiazol-2-yl)-2,5-diphenyltetrazolium bromide (MTT) 3-(4,5-dimethylthiazol-2-yl)-2,5-diphenyltetrazolium bromide assay with L-929 fibroblasts cell as a model cell. L-929 cells were cultured (5,000) and placed in an incubator under saturated humid conditions at 37°C and 5% CO_2_ in each 24-well plates for 24 h cultivation. Our nanocarriers (A_100_N_150_/Fe_3_O_4_-NH_2_ or A_100_N_150_/Fe_3_O_4_-NH_2_/R6G) were mixed with PBS solution and sterilized through a 0.22 μm membrane filter. Thus, the sterilized solution was mixed with DMEM and denoted as the A_100_N_150_/Fe_3_O_4_-NH_2_-contained or A_100_N_150_/Fe_3_O_4_-NH_2_/R6G-contained medium. For toxicity studies, L-929 cells were incubated with those two mediums for 3 days. Then the control groups, pure DMEM (negative control) or 5% dimethyl sulfoxide (DMSO) in DMEM (positive control) were studied. After culturing, the resulting blue-purple crystals were dissolved in pure DMSO for 15 min, so that the cell number could be calculated by enzyme-linked immunosorbent assay (ELISA) (Sunrise, Tecan Group Ltd., Zürich, Switzerland).

## Results and discussion

### Synthesis and molecular characterization of P(AA-b-NIPAAm-b-AA)

In this study, RAFT polymerization was introduced to synthesize the dual functional tri-block copolymers, PNIPAAm with AA segments on both polymer-chain ends, denoted as P(AA-b-NIPAAm-AA). The molecular weight of tri-block copolymer was calculated by Equation 4 as below:

(4) $$\mathrm{Mn}={\mathrm{Mn}}_{\mathrm{PAA},\mathrm{GPC}}+{\mathrm{Mn}}_{\mathrm{PNIPAAm},\mathrm{NMR}}$$

where Mn refers to the number average molecular weight of copolymer, and Mn_PAA,GPC_ stands for the number average molecular weight of PAA estimated via GPC. Mn_PNIPAAm,NMR_ represents the number average molecular weight of PNIPAAm chains via NMR analysis. The molecular weight, Mn_exp_, calculated by Equation 4 seems not equal to the experimental molecular weight estimated by GPC, Mn_GPC_, as shown in Table 1. Since the standard of GPC was poly(methylacrylic acid) (PMAA), the structural differences between PMAA and tri-block copolymers led to the deviation. Since S,S′-bis(α,α′-dimethyl-α″-acetic acid)trithiocarbonate was employed as CTA for living polymerization, narrow molecular weight distribution (Mw/Mn = 1.27) was obtained, and the repeating unit ratio to feeding ratio ([AA] /[NIPAAm] = 1/1.45) estimated by NMR analysis was close to the feeding ratio as shown in Table 1.

**Table 1:** Molecular characterization of P(AA-b-NIPAAm-AA)

**Sample Code**	**[AA]**_**0 **_**/[NIPAAm]**_**0**_	**[AA]**_**NMR **_**/[NIPAAm]**_**NMR**_	**Mn**_**GPC**_^**a**^	**Mn**^**b**^	**PDI**^**c**^
A_100_N_150_	1/1.5	1/1.45	48,572	30,997	1.27

^a^The experimental molecular weight estimated by GPC; ^b^the molecular weight calculated by Eqation 4; ^c^polydispersity index (PDI) = Mw/Mn, from GPC.

### LCST of P(AA-b-NIPAAm-b-AA)

**Figure 2:**
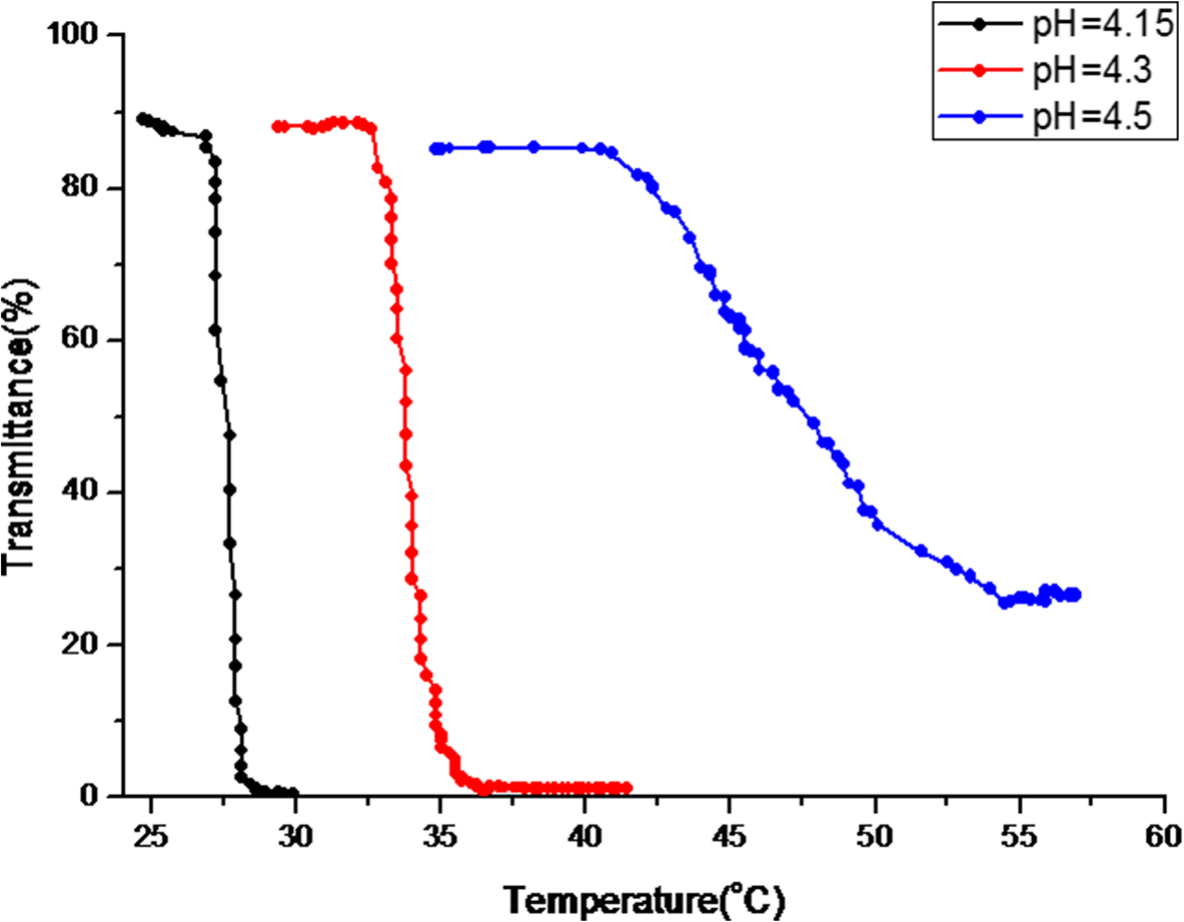
**UV/Vis transmittance spectrum of P(AA-b-NIPAAm-b-AA) dissolved in the various pH value buffer solutions at various temperatures.** The LCSTs were: 27°C, pH = 4; 32.6°C, pH = 4.3; and 40.7°C, pH = 4.5, respectively.

The synthesized RAFT-polymerized PNIPAAm bearing carboxylic acid functional groups on both sides of the polymer chain was denoted as P(AA-b-NIPAAm-b-AA). P(AA-b-NIPAAm-b-AA) was dissolved in various pH value solutions with diluted NaOH solution for adjustment. The LCST was determined by measuring the transmittance of the latex solution at 480 nm and different temperatures. As shown in Figure 2, the LCST of P(AA-b-NIPAAm-b-AA) was increasing as the pH value increased. Since the tri-block copolymer would become much more hydrophilic owing to the deprotonation of carboxylic acid groups at high pH values, the LCST increases from 27°C to 40.7°C as pH increases from 4.15 to 4.5.

### FT-IR analysis

**Figure 3:**
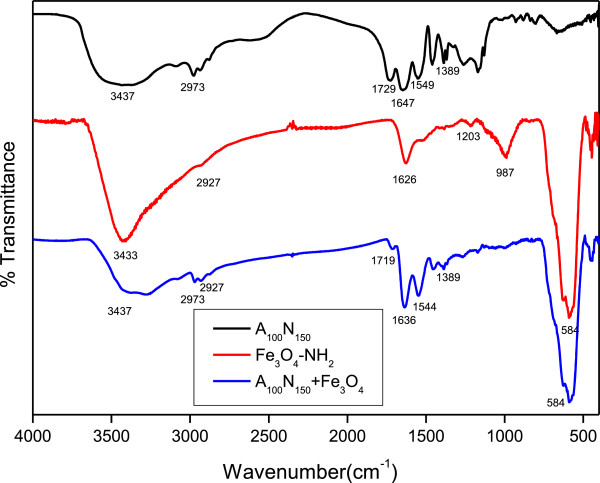
**FT-IR spectra of A**_**100**_**N**_**150**_**, Fe**_**3**_**O**_**4**_**-NH**_**2 **_**and A**_**100**_**N**_**150**_ **+ Fe**_**3**_**O**_**4**_**-NH**_**2**_**, respectively.**

Surface functional groups of our tri-block copolymer and nanoparticles were analyzed via FT-IR as shown in Figure 3. The absorption peaks of A_100_N_150_ at 3,437, 2,973, 1,729, 1,647, 1,549, and 1,389 cm^-1^ represent N-H stretching of amide groups, C-H stretching, C = O stretching of carboxylic acids, C = O stretching of amides, C-N stretching, and C-H stretching of CH(CH)_3_, respectively. The peaks of Fe_3_O_4_-NH_2_ at 3,433, 2,927, 1,626, 1,203, 987, and 584 cm^-1^, corresponding to N-H stretching, C-H stretching, C-N stretching, Fe-O-Si bond stretching, and Fe-O bond vibration, respectively, demonstrated the surface modification of aminosilane on Fe_3_O_4_ nanoparticles. After crosslinking reaction via EDC/NHS activation between A_100_N_150_ and Fe_3_O_4_-NH_2_, the proportion of carboxylic acids (1,719 cm^-1^) among C = O stretching reduced significantly and also the adsorption peak of Fe-O, 584 cm^-1^ was detected, as shown in the A_100_N_150_/Fe_3_O_4_.

### Surface potential

The surface potential of sub-micron particles was determined as 0.002 via zeta potential analyzer which indicated that the tri-block copolymer, A_100_N_150_, was closed to the isoelectric point at pH = 4.5 and 60°C. Owing to the hydrophilic property of PAA segments, which provided the stability to the extreme hydrophobic PNIPAAm segments, the uncharged sub-micron particles could disperse well in an aqueous solution.

### Morphology observation

**Figure 4:**
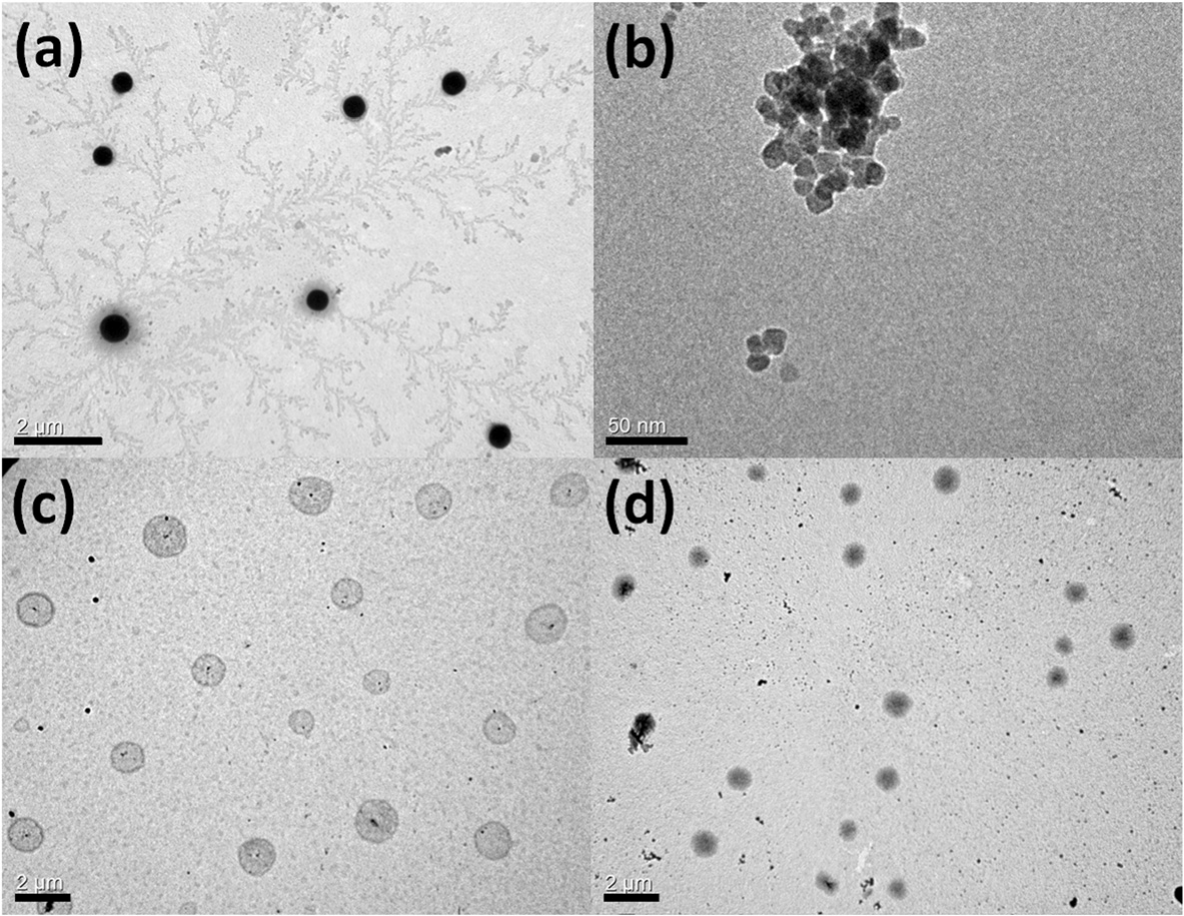
**TEM images of self-assembled sub-micron particles. (a)** A_100_N_150_ at pH = 4.5, 60°C, (400 to 600 nm); **(b)** Fe_3_O_4_-NH_2_, (15 nm); **(c)** A_100_N_150_/Fe_3_O_4_-NH_2_ at 60°C, (550 to 1,000 nm); and **(d)** A_100_N_150_/Fe_3_O_4_-NH_2_ at 25°C, (600 to 1,300 nm).

Acrylic acid was copolymerized with NIPAAm to introduce the hydrophilic segments in copolymers. According to Figure 2, the hydrophility of the tri-block copolymer could be adjusted via changing of the pH value and the temperature of the system. While the system temperature was above the LCST, self-assembling phenomenon was triggered by the phase transition of PNIPAAm segments. Thus, the 400 to 600 nm, core-shell, self-assembled sub-micron particles composed of hydrophobic PNIPAAm core and hydrophilic PAA shell could be observed at pH = 4.5 and 60°C as shown in Figure 4a. The surface-modified iron oxides with 15 nm (Figure 4b) were introduced into the self-assembling sub-micron particle system in order to not only fix the spherical nanostructure at various temperatures but also render the stimuli-response resource for magnetic-triggered release. Figure 4c suggested that there were micro phase separations consisting of the hydrophobic PNIPAAm and hydrophilic PAA in the spherical nanostructure, where Figure 4d exhibited the homogeneous morphology owing to the hydrophilic transition of PNIPAAm. It is obvious whether the temperature was above LCST or not, the magnetic sub-micron particles, A_100_N_150_/Fe_3_O_4_-NH_2_, as shown in Figure 4c,d could maintain its nanostructure because of the high degree of crosslinking and the particle size of A_100_N_150_/Fe_3_O_4_-NH_2_ increased only slightly after swelling.

### Preparation of R6G-loaded magnetic nanocarriers via swelling method

**Figure 5:**
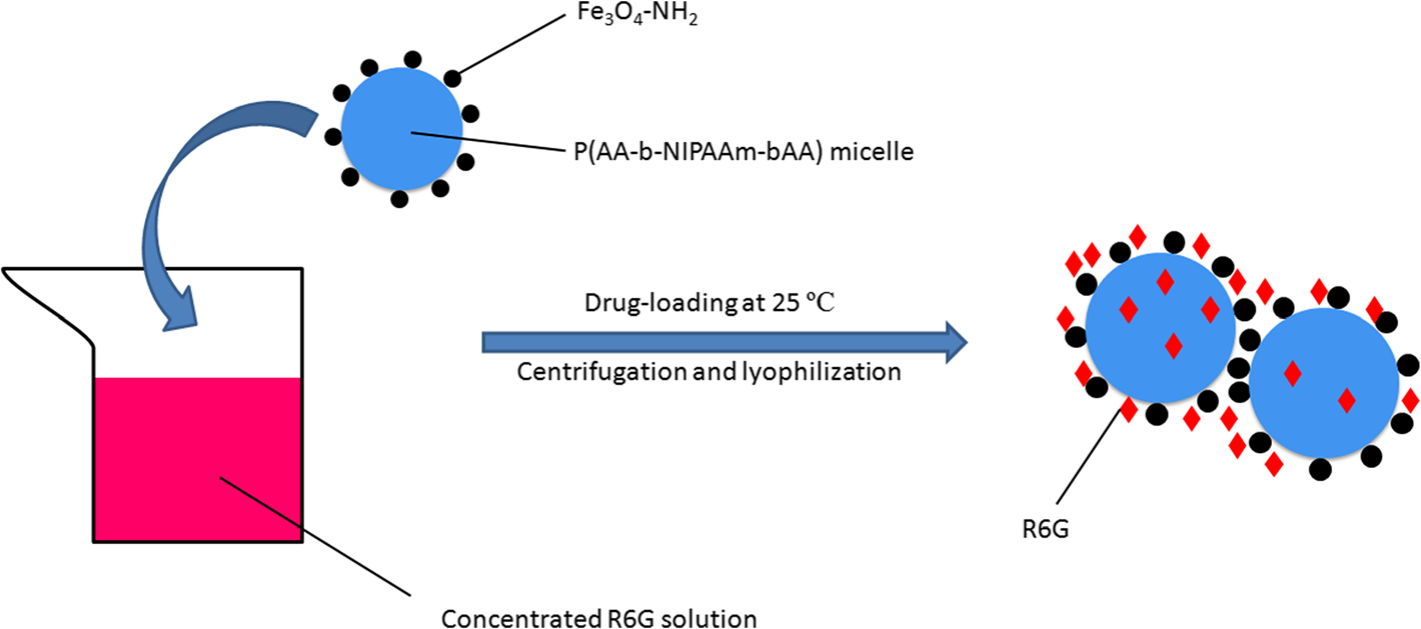
Schematic models of the nanostructure for the R6G-loading process.

**Figure 6:**
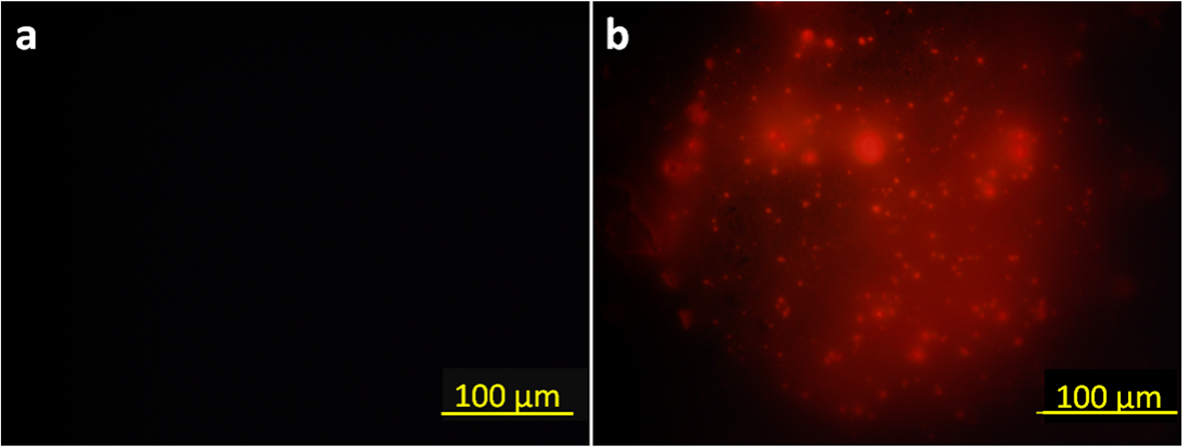
**Fluorescence microscopy images of nanocarriers at 25°C. (a)** Magnetic-nanocarriers, A_100_N_150_/Fe_3_O_4_-NH_2_, and **(b)** R6G-loaded magnetic nanocarriers, A_100_N_150_/Fe_3_O_4_-NH_2_/R6G.

In our previous study [21], R6G molecules had shown strong interaction with the tri-block copolymer, P(AA-b-NIPAAm-b-AA), thus the characteristic would be utilized for drug encapsulation. It is obvious that R6G not only could diffuse into the magnetic nanocarriers but also participate on the surface during the loading process as shown in Figure 5. The swelling ratio of magnetic nanocarriers was 150%, lower than that of pure PNIPAAm (500% to 600%), after loading and centrifugation process. The R6G-loaded nanocarriers were then lyophilized for the further experiments. Meanwhile, the encapsulation efficiency (EE) and drug loading content (DLC) were determined by UV/Vis as 99.01% and 371.3 mg/g, respectively.Figure 6 shows the optical images of diluted nanocarrier solutions taken from the fluorescent microscopy. Because of the limitation of observation scale, it was difficult to identify whether the R6G was encapsulated into the nanocarriers or deposited on the surface. However, comparing Figure 6a,b, R6G-loaded nanocarriers indeed exhibit the fluoresce capability, and only the bright dots could be detected without aggregation of the brightness. This implied the complete encapsulation of R6G molecules in the nanocarriers.

### Cell line and cytotoxicity analysis

**Figure 7:**
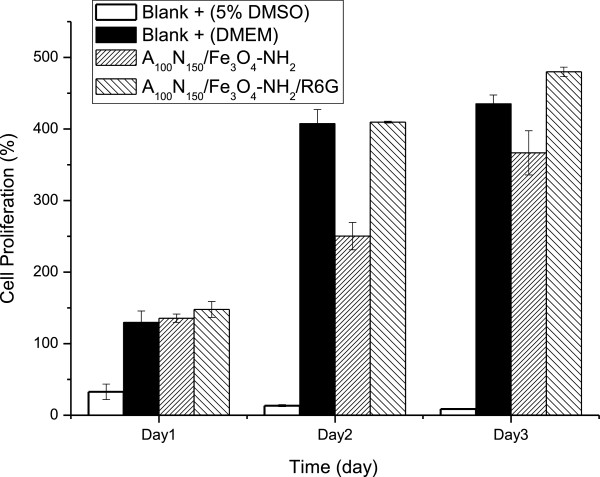
**MTT analysis of A**_**100**_**N**_**150**_**/Fe**_**3**_**O**_**4**_**-NH**_**2 **_**and A**_**100**_**N**_**150**_**/Fe**_**3**_**O**_**4**_**-NH**_**2**_**/R6G using L-929 cells.**

MTT assay was applied to determine the cellular cytotoxicity of our nanocarriers. Figure 7 shows the cell proliferation results of control and sample groups incubated with the L-929 cells. Pure DMEM and 5% DMSO in DMEM were used as the material of negative and positive control groups, respectively, and magnetic nanocarriers and R6G-loaded nanocarriers were served as those of the sample groups. It could be found that L-929 cells in both of the sample groups remained growing during the days of incubation, and it confirmed that our nanocarriers have no cytotoxicity. As a matter of fact, the magnetic nanocarriers have potentials for biomedical applications.

### Controlled release with various pH values and temperatures

**Figure 8:**
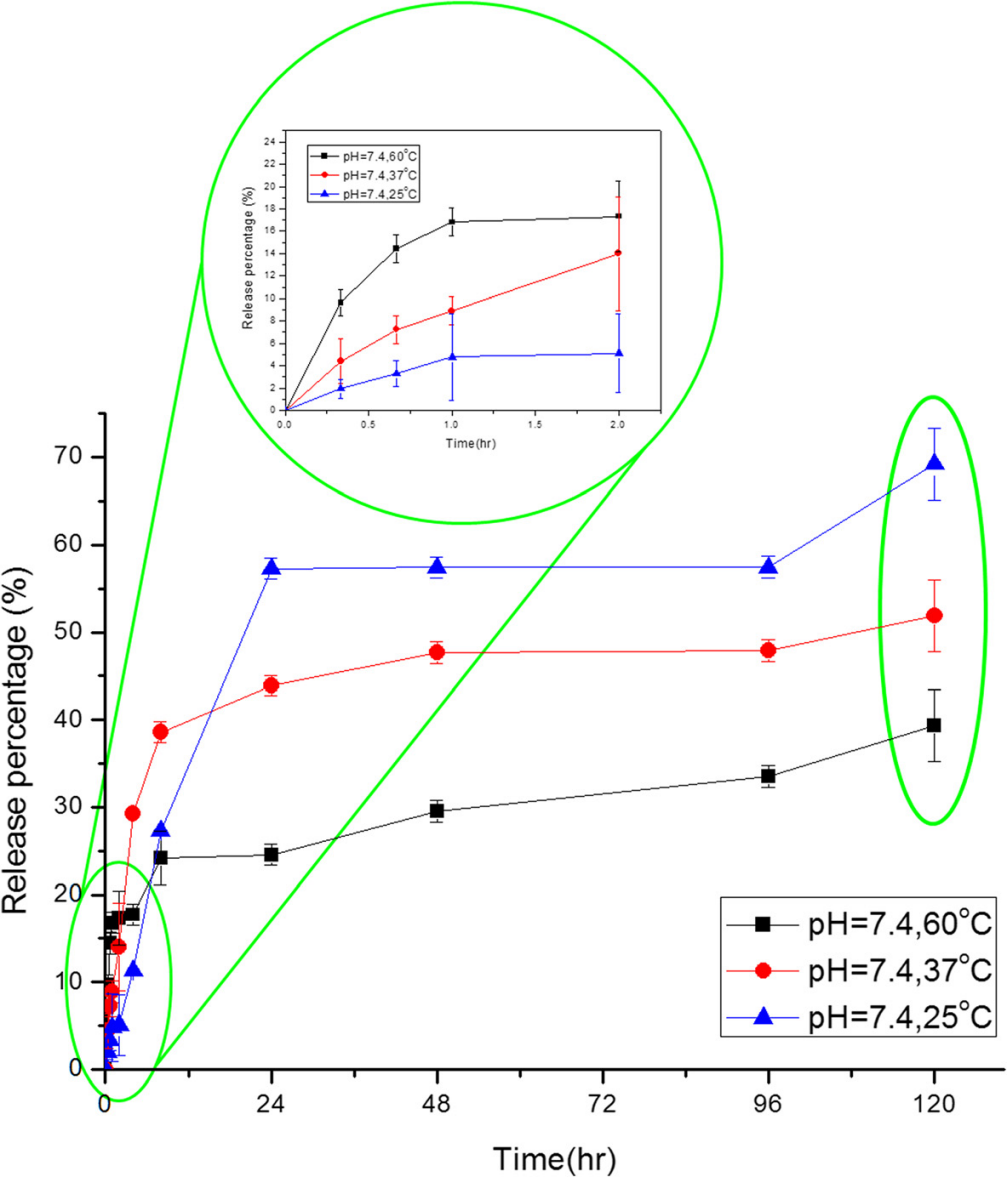
The controlled release behaviors of the magnetic nanocarriers with different temperatures at pH = 7.4.

**Figure 9:**
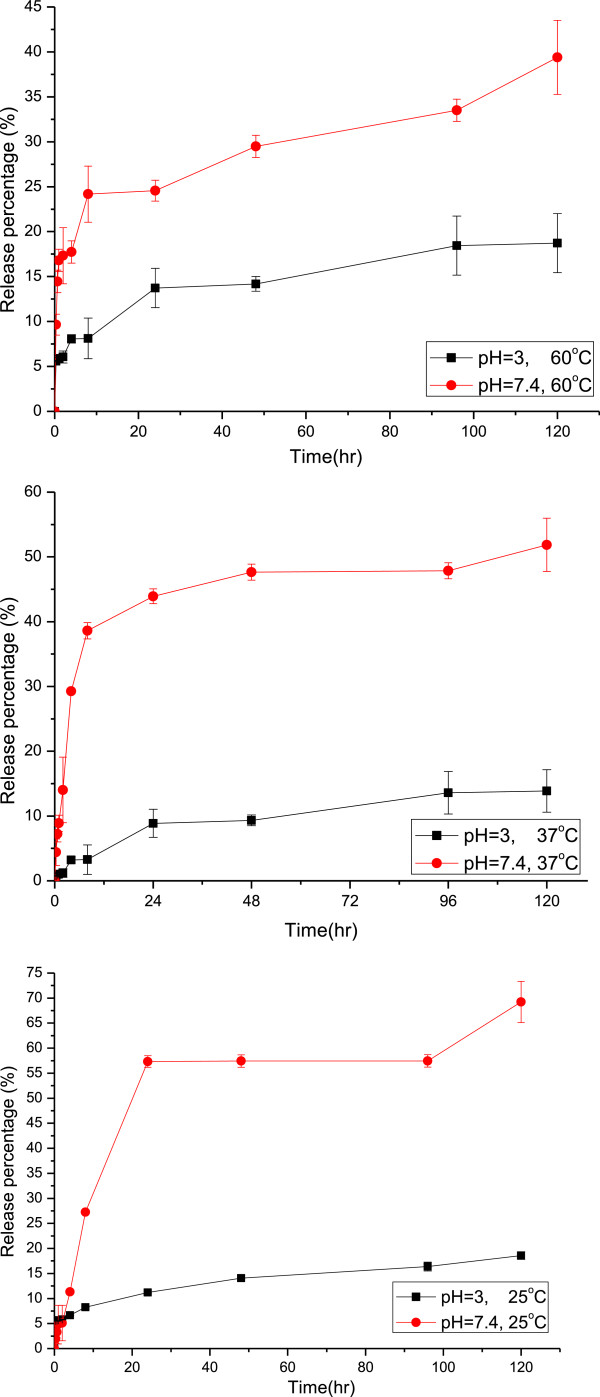
The comparison of drug released behaviors at pH = 3 or 7.4 with various temperatures.

The stimuli-triggered release behaviors of the R6G-loaded nanocarriers were studied via immersing the lyophilized nanocarrier powders in various buffer solutions at different temperatures. Figure 8 exhibits the influence of temperature for the controlled release system at pH = 7.4. It was found that the release behavior could be distinguished in two periods, the initial period (0 to 2 h) and the final one (2 to 120 h). Temperature has dominated the system in the beginning of the controlled release, which showed that the amount of release was proportional to the temperature. As aforementioned, R6G was loaded both in the core of magnetic nanocarrier and on the surface of it. At first, the R6G bonded on the surface dissolved and diffused into the buffer solution, so that the release amount was increasing as the temperature rose due to the higher diffusion rate. As time passed, R6G encapsulated in the core of the nanocarriers gradually diffused out and the diffusion rate inside the nanocarriers has dominated the release process. High temperature suggested a serious shrinkage of PNIPAAm as a physical barrier of diffusion. As a result, the final release percentage decreased as the temperature increased.Figure 9 illustrated the comparison of release percentages at pH = 3 and 7.4 with various temperatures. Actually, the nanocarrier extremely aggregated at pH = 3 during the controlled release owing to the strong intermolecular hydrogen bonding composed of the protonated carboxylic acid groups and the amide groups in PNIPAAm. It is obvious that the pH value strongly influenced the release behavior at various temperatures. Because of the deprotonation of the carboxylic acid groups on the surface of nanocarriers at pH = 7.4, the carriers then became more hydrophilic than at pH = 3 so that the diffusion rate of R6G and the final release percentage were both increased.

### Magnetic-triggered release

**Figure 10:**
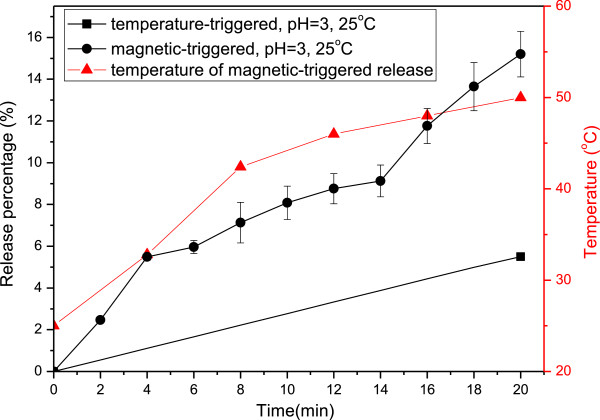
The comparison of stimuli-trigged released behaviors with and without HFMF applied.

The smart controlled release of magnetic nanocarriers was employed via HFMF. Since the surface-modified iron oxide, Fe_3_O_4_-NH_2_, was introduced in order to maintain the spherical structure of PNIPAAm-based sub-micron particles, the remote control of magnetic nanocarriers for particle movement, controlled release and thermal treatment became possible owing to the hyperthermia effect. According to Figure 10, the release percentage of the magnetic-triggered one was three times higher than that of the temperature-triggered one for only 20 min. Meanwhile, the bulk solution temperature of HFMF-applied controlled release could be increased to 50°C due to the hyperthermia effect. It is expected that the local temperature of magnetic nanocarriers should be higher than 50°C so that the rapid-response release could be obtained.

## Conclusions

Polymer-framed, stimuli-response sub-micron particles were designed and prepared with magnetic iron oxides as intelligent multifunctional nanocarriers. RAFT polymerization was first employed for the P(AA-b-NIPAAm-b-AA) tri-block copolymer synthesis in order to obtain the well-defined self-assembling sub-micron particles. With EDC/NHS crosslinking method, Fe_3_O_4_-NH_2_ bonded with the tri-block copolymer micelle could not only provide the magnetically resource but also prevents the collapses of the spherical structure. The magnetic-triggered nanocarriers did not display cytotoxicity in L-929 fibroblasts, which provided the benefits for medical therapy like oral dosing. R6G was able to be encapsulated in the core as well as on the surface of nanocarriers due to the strong interaction between R6G and the polymers. The stimuli-response release behavior reveals that the intelligent multifunctional nanocarriers were highly pH and magnetic sensitive owing to the iron oxides and the functional groups (-COOH and -NH_2_) on its surface. Since the amount of release increased as the pH value increased, the nanocarrier has great potential for therapeutic application to intestinal illness with the demand of low leakage in stomach where the pH was in the range of 2 to 3. Moreover, the multifunctional nanocarriers exhibit three times of the release percentage under high-frequency magnetic field treatment in a short period (temperature above 50°C), which could acquire rapid and accurate therapy in practical applications. On the whole, there is a great potential of using the intelligent, multifunctional nanocarriers in biomedical targeting therapy especially for intestinal sickness.

## Competing interests

The authors declare that they have no competing interests.

## Authors’ contributions

CYK, CFL, MSW, TYL, and WYC had conceived and designed the experiments. CYK and AH, performed the experiments. CYK, AH, and WYC contributed ideas and material analyses. CYK wrote the manuscript. All authors read and approved the final manuscript.

## Authors’ information

CYK is a PhD students at National Taiwan University. AH is a PhD students at National Taiwan University of Science and Technology. CFL holds a professor position at Chia Nan University of Pharmacy and Science. MSW is a researcher at National Taipei University of Technology. TYL holds an assistant professor position at Ming Chi University of Technology. WYC holds a professor position at National Taiwan University.

## References

[B1] Meier W Polymer nanocapsules ChSRv 2000 29 295 303

[B2] Sun P Zhang Y Shi L Gan Z Thermosensitive nanoparticles self-assembled from PCL-b-PEO-b-PNIPAAm triblock copolymers and their potential for controlled drug release Macromol Biosci 2010 10 621 631 10.1002/mabi.200900434 20166233

[B3] Torchilin V Tumor delivery of macromolecular drugs based on the EPR effect Adv Drug Deliv Rev 2011 63 131 135 10.1016/j.addr.2010.03.011 20304019

[B4] Ferguson CJ Hughes RJ Pham BTT Hawkett BS Gilbert RG Serelis AK Such CH Effective ab initio emulsion polymerization under RAFT control Macromolecules 2002 35 9243 9245 10.1021/ma025626j

[B5] Du JZ Sun TM Song WJ Wu J Wang J A tumor-acidity-activated charge-conversional nanogel as an intelligent vehicle for promoted tumoral-cell uptake and drug delivery Angew Chem Int Ed Engl 2010 49 3621 3626 10.1002/anie.200907210 20391548

[B6] Lefaux CJ Zimberlin JA Dobrynin AV Mather PT Polyelectrolyte spin assembly: influence of ionic strength on the growth of multilayered thin films J Polym Sci Part B: Polym Phys 2004 42 3654 3666 10.1002/polb.20209

[B7] Liu TY Hu SH Liu KH Liu DM Chen SY Study on controlled drug permeation of magnetic-sensitive ferrogels: effect of Fe3O4 and PVA J Control Release 2008 126 228 236 10.1016/j.jconrel.2007.12.006 18237812

[B8] Heskins M Guillet JE Solution properties of Poly(N-isopropylacrylamide) J MACROMOL SCI A-CHEM 1968 2 1441 1455 10.1080/10601326808051910

[B9] Balamurugan SS Bantchev GB Yang Y McCarley RL Highly water-soluble thermally responsive Poly(thiophene)-Based brushes Angew Chem 2005 117 4950 4954 10.1002/ange.200500867 15988778

[B10] Caruso F Hollow capsule processing through colloidal templating and self-assembly Chem Eur J 2000 6 413 419 10.1002/(SICI)1521-3765(20000204)6:3<413::AID-CHEM413>3.0.CO;2-9 10747405

[B11] Chiang WH Huang WC Chang CW Shen MY Shih ZF Huang YF Lin SC Chiu HC Functionalized polymersomes with outlayered polyelectrolyte gels for potential tumor-targeted delivery of multimodal therapies and MR imaging J Control Release 2013 168 280 288 10.1016/j.jconrel.2013.03.029 23562635

[B12] Hu SH Liao BJ Chiang CS Chen PJ Chen IW Chen SY Core-shell nanocapsules stabilized by single-component polymer and nanoparticles for magneto-chemotherapy/hyperthermia with multiple drugs Adv Mater 2012 24 3627 3632 10.1002/adma.201201251 22689346

[B13] Huang H-Y Hu S-H Chian C-S Chen S-Y Lai H-Y Chen Y-Y Self-assembling PVA-F127 thermosensitive nanocarriers with highly sensitive magnetically-triggered drug release for epilepsy therapy in vivo JMCh 2012 22 8566

[B14] Liu T-Y Hu S-H Liu D-M Chen S-Y Chen IW Biomedical nanoparticle carriers with combined thermal and magnetic responses Nano Today 2009 4 52 65 10.1016/j.nantod.2008.10.011

[B15] Liu T-Y Hu S-H Liu K-H Shaiu R-S Liu D-M Chen S-Y Instantaneous drug delivery of Magnetic/Thermally sensitive nanospheres by a high-frequency magnetic field Langmuir 2008 24 13306 13311 10.1021/la801451v 18954093

[B16] Liu T-Y Liu K-H Liu D-M Chen S-Y Chen IW Temperature-sensitive nanocapsules for controlled drug release caused by magnetically triggered structural disruption Adv Funct Mater 2009 19 616 623 10.1002/adfm.200801304

[B17] Ruhland TM Reichstein PM Majewski AP Walther A Muller AH Superparamagnetic and fluorescent thermo-responsive core-shell-corona hybrid nanogels with a protective silica shell J Colloid Interface Sci 2012 374 45 53 10.1016/j.jcis.2012.01.028 22364711

[B18] Pauzauskie PJ Radenovic A Trepagnier E Shroff H Yang P Liphardt J Optical trapping and integration of semiconductor nanowire assemblies in water Nat Mater 2006 5 97 101 10.1038/nmat1563 16429143

[B19] Lai JT Filla D Shea R Functional polymers from novel carboxyl-terminated trithiocarbonates as highly efficient RAFT agents Macromolecules 2002 35 6754 6756 10.1021/ma020362m

[B20] Sahoo B Devi KS Banerjee R Maiti TK Pramanik P Dhara D Thermal and pH responsive polymer-tethered multifunctional magnetic nanoparticles for targeted delivery of anticancer drug ACS Appl Mater Interfaces 2013 5 3884 3893 10.1021/am400572b 23551195

[B21] Kuo C-Y Wang Y-C Lee C-F Chiu W-Y A novel route for preparation of multifunctional polymeric nanocarriers for stimuli-triggered drug release J Polym Sci Part A: Polym Chem 2014 52 561 571

